# Developmental Dysplasia of the Hip: Clinical Predictors and the Impact of a Screening Protocol

**DOI:** 10.7759/cureus.114743

**Published:** 2026-08-18

**Authors:** Lucinda Amorim Delgado, Liane Moreira, Liliana Carvalho de Sousa, Nuno Henrique Afonso, Inês Lemos Martins, Margarida Peixoto, Alexandra Pinto, André Coelho Almeida

**Affiliations:** 1 Pediatrics, Unidade Local de Saúde do Alto Ave, Guimarães, PRT; 2 Family Medicine, Unidade de Sáude Familiar Bem Viver, Marco de Canaveses, PRT; 3 Neonatology, Unidade Local de Saúde do Alto Ave, Guimarães, PRT

**Keywords:** clinical protocol, developmental dysplasia of the hip, risk factors, screening, ultrasound

## Abstract

Background

Developmental dysplasia of the hip (DDH) includes a spectrum of hip abnormalities that can cause significant functional impairment if not diagnosed early. Currently, no universal consensus exists regarding the optimal screening strategy. In March 2020, a new DDH screening protocol was implemented at a hospital in northern Portugal. This study aimed to assess the prevalence of DDH, identify associated risk factors and physical examination findings, and evaluate clinical practice changes post-protocol.

Methodology

We conducted a retrospective, observational, cohort study analyzing hip ultrasounds from August 2017 to August 2024 in infants up to 12 months of age. The cohort was divided into Pre-Protocol and Post-Protocol groups.

Results

The study included 816 infants (402 (49.3%) Pre-Protocol, 414 (50.7%) Post-Protocol), and 38 cases (4.7%) of DDH were identified. Positive Ortolani (odds ratio (OR) = 53.2) and Barlow (OR = 13.2) tests, as well as congenital foot anomalies (OR = 5.4), were significantly associated with DDH. Following protocol implementation, referrals for prematurity decreased significantly (55 (13.7%) vs. 13 (3.1%), p < 0.001), while referrals for breech presentation (142 (35.3%) vs. 182 (44.0%), p = 0.012) and hip click (55 (13.7%) vs. 87 (21.0%), p = 0.006) increased.

Conclusions

Protocol implementation led to optimized referrals, reducing low-yield screenings for prematurity while increasing detection efforts for breech presentation. However, referrals for hip clicks increased, suggesting a need for further education. Physical examination remains the cornerstone of screening.

## Introduction

Developmental dysplasia of the Hip (DDH) is a common condition affecting the pediatric population, including a broad spectrum of progressive structural abnormalities ranging from mild acetabular dysplasia and subluxation to complete femoral head dislocation. Pathophysiologically, it involves abnormal development of the acetabulum and femoral head. Clinically, in newborns, it can present with subtle signs such as limitation of hip abduction, asymmetric thigh or gluteal folds, apparent limb length discrepancy (Galeazzi sign), or positive dynamic instability maneuvers (Ortolani and Barlow tests) [1]. If left undiagnosed or untreated in infancy, DDH can lead to devastating long-term consequences, including persistent gait abnormalities, chronic pain, and early-onset hip osteoarthritis in young adulthood [1]. The early detection of this condition is, therefore, a priority in neonatal and pediatric care.

Despite the clinical importance of DDH, a universal consensus on the optimal screening strategy remains elusive. Globally, and particularly across Europe, screening practices vary significantly [2]. Several countries, such as Germany, Austria, and Italy, have implemented universal ultrasound screening programs for all newborns, aiming to minimize late-detected cases and subsequent surgical interventions [2,3]. In contrast, healthcare systems in the United Kingdom, North America, and Australia, often guided by institutions such as the American Academy of Pediatrics, the National Institute for Health and Care Excellence [4], and the U.S. Preventive Services Task Force [5], advocate for a selective screening approach. This selective strategy targets only infants exhibiting abnormal physical examination findings or specific risk factors, such as breech presentation or a positive family history. While universal screening may further reduce late diagnoses, it is associated with higher initial costs and potential overtreatment, whereas selective screening raises concerns about missed diagnoses in infants without apparent risk factors [6].

Regardless of the screening method employed, early and accurate diagnosis is the fundamental prerequisite for achieving optimal clinical outcomes and minimizing the risk of gait abnormalities, chronic pain, and early-onset hip osteoarthritis in adulthood [1,6]. 

At a hospital in northern Portugal, an institutional screening protocol for DDH was implemented in March 2020 [7]. The primary objective of this protocol was to standardize referral practices and allocate resources to infants at highest risk of pathology.

According to the institutional protocol, all newborns undergo a physical examination. Infants with a normal examination but presenting specific risk factors, namely, breech presentation, history of oligohydramnios, first-degree family history of DDH, congenital foot deformities, congenital torticollis, or polymalformative syndromes, are systematically referred for a hip ultrasound at six weeks of age. Conversely, infants presenting with altered physical examination findings, such as a positive Ortolani sign, asymmetric gluteal folds, limited hip abduction, or limb length discrepancy, prompt an urgent orthopaedic evaluation. Cases with equivocal or inconclusive physical examinations are re-evaluated after two weeks, and an ultrasound is requested if the findings persist.

The objectives of this study were to characterize the population screened at this hospital, determine the prevalence of DDH, and assess the predictive value of selected physical examination findings. Furthermore, we aimed to assess the impact of the 2020 protocol implementation on clinical practice by comparing referral patterns and risk factor profiles before and after its introduction.

## Materials and methods

Study design and ethics

This was a retrospective, observational cohort study. Data were anonymized to strictly maintain patient confidentiality in accordance with ethical standards.

Population and setting

The study was conducted at the pediatric and neonatology departments of a hospital in northern Portugal. We reviewed the clinical records of all infants up to 12 months who underwent hip ultrasound for DDH screening over a seven-year period, from August 2017 to August 2024. Inclusion criteria were infants aged up to 12 months who underwent hip ultrasound for DDH screening between August 2017 and August 2024 and had available clinical and ultrasound records. Exclusion criteria were age over 12 months, hip ultrasound performed outside the defined study period, ultrasound performed for indications other than DDH screening or for follow-up of previously diagnosed DDH, and insufficient clinical or ultrasound data for analysis.

Study groups

To evaluate the impact of the new screening guidelines (Table 1) [7], the study population was divided into two distinct chronological groups: the Pre-Protocol Group, which included infants screened between August 2017 and February 2020, and the Post-Protocol Group, which included infants screened between March 2020 and August 2024. Importantly, before March 2020, there was no formal or standardized DDH screening protocol implemented at the hospital. During the Pre-Protocol period, clinical management, screening indications, and referrals for hip ultrasound were based on the individual clinical judgment and personal experience of the attending pediatricians and family physicians. The implementation of the March 2020 protocol represented the first standardized institutional guideline designed to regulate and unify DDH screening indications and referral pathways across departments.

**Table 1:** Institutional screening protocol for developmental dysplasia of the hip. DDA: developmental dysplasia of the hip; PHC: primary healthcare; NCHYP: National Child and Youth Health Program

Hip physical examination	Risk factors for DDH	Clinical management and examinations	Referral/Follow-up
Abnormal/With alterations (Positive Ortolani, asymmetry of gluteal folds, limitation in hip abduction, or limb length discrepancy)	Present or absent	Physical examination with alterations in the newborn	Urgent collaboration with Orthopedics
Equivocal or inconclusive	Present or absent	Re-evaluation of the newborn after 2 weeks. If the hip examination remains inconclusive or doubtful, request a hip ultrasound at 6 weeks	Guidance based on ultrasound results or persistent clinical doubt
Normal	Present (breech presentation, history of oligohydramnios, family history (first-degree relative), congenital foot deformities, congenital torticollis or polymalformative syndrome)	Request hip ultrasound performed at 6 weeks and re-evaluation in Neonatology consultation.	If normal ultrasound: Maintain follow-up in PHC according to the periodicity stipulated in the NCHYP. If ultrasound abnormalities: Referral to Orthopedics
Normal	Absent	Maintain routine surveillance in the newborn	Follow-up in PHC according to the periodicity stipulated in the NCHYP

Data collection and variables

Data were extracted from digital clinical records. We collected demographic variables (age, sex), clinical history (family history of DDH, pregnancy details, breech presentation, oligohydramnios), and physical examination findings. The diagnosis of DDH was confirmed based on ultrasound reports. All hip ultrasounds were performed and interpreted by radiologists trained in hip ultrasonography. The ultrasonographic evaluation and classification of DDH severity were strictly based on the standardized Graf method.

Statistical analysis

Statistical analysis was performed using SPSS Statistics for Windows, Version 25.0 (IBM Corp., Armonk, NY, USA). Categorical variables were described using frequencies and percentages. Continuous variables were described using medians and interquartile ranges (IQRs) due to non-normal distribution. To identify risk factors associated with DDH, we calculated adjusted odds ratios (ORs) with 95% confidence intervals (CIs). Comparisons between the Pre-Protocol and Post-Protocol groups were made using the chi-square test or Fisher’s exact test, as appropriate. To identify independent risk factors associated with DDH, a multivariable logistic regression model was used to calculate adjusted ORs with 95% CIs. Variables demonstrating clinical relevance or statistical significance in univariate analysis were entered into the model, and a backward stepwise selection method was applied to control for confounding variables. A p-value of less than 0.05 was considered statistically significant.

## Results

Demographic characteristics

A total of 816 infants met the inclusion criteria and were included in the analysis. The population was nearly evenly split between the two study periods, with 402 (49.3%) infants in the Pre-Protocol Group and 414 (50.7%) infants in the Post-Protocol Group. The gender distribution showed a slight female predominance (427 female (52.3%) vs. 389 male (47.7%)).

The indications for ultrasound referral in the screened population encompassed a wide range of risk factors and clinical findings. The most frequent reason for referral was third-trimester breech presentation (324 (39.7%)), followed by hip clicks (142 (17.4%)), asymmetric skin folds (128 (15.7%)), limited hip abduction (128 (15.7%)), and a first-degree family history of DDH (113 (14.1%)). Other notable reasons included twin pregnancy (58 (7.1%)), a second-degree family history (47 (5.9%)), positive Ortolani (34 (4.2%)) and Barlow (14 (1.7%)) maneuvers, congenital torticollis (27 (3.3%)), congenital foot anomalies (17 (2.1%)), sacrococcygeal double folds (18 (2.2%)), oligohydramnios (8 (1.0%)), positive Galeazzi sign (8 (1.0%)), limb length discrepancy (7 (0.9%)), and polymalformative syndromes (5 (0.6%)).

The median age at the time of the first screening ultrasound was eight weeks (IQR = 8). A small subset of patients (62 (7.6%)) required a repeated ultrasound, which occurred at a median age of 18 weeks.

Prevalence and clinical features of developmental dysplasia of the hip

DDH was diagnosed in 38 of the 816 screened infants, yielding a prevalence rate of 4.7%. Consistent with the literature, the condition was more common in females (23 (60.5%)) compared to males (15 (39.5%)). Regarding laterality, the left hip was most frequently affected (21 (55.3%)), followed by bilateral involvement (11 (28.9%)) and the right hip (6 (15.8%)).

Analysis of predictors

The study identified strong associations between specific physical examination findings and the presence of DDH. The most significant predictor was a positive Ortolani test, which was associated with an adjusted OR of 53.2 (95% CI: 21.6-131.0; p = 0.000). The Barlow test also showed a strong predictive value (OR = 13.2; 95% CI: 2.6-67.4; p = 0.002). Among musculoskeletal anomalies, congenital foot anomalies were significantly associated with DDH (OR = 5.4; 95% CI: 1.1-25.8; p = 0.034).

Furthermore, the data revealed that there were no diagnosed cases of DDH associated with lesser-established risk factors when these were present in isolation. Specifically, there were zero cases of DDH among infants referred solely due to an isolated second-degree family history, isolated prematurity, or an isolated hip click.

Impact of protocol implementation

The comparison between the Pre-Protocol and Post-Protocol periods revealed significant shifts in referral patterns (Table 2). One of the most notable changes was the reduction in referrals for prematurity. In the Pre-Protocol period, 55 (13.7%) infants were referred primarily for this reason, whereas in the Post-Protocol period, this number dropped to 13 (3.1%) infants (p = 0.000). This suggests a successful discontinuation of routine screening for premature infants without other risk factors.

**Table 2:** Changes in referral patterns: comparison of Pre- and Post-Protocol periods. FH: family history; RF: risk factors

RF or physical examination signs	Before (n = 402)	After (n = 414)	P-value
Female sex	205 (51.0%)	222 (53.6%)	0.452
First-grade FH	49 (12.6%)	64 (15.5%)	0.244
Second-grade FH	17 (4.4%)	30 (7.2%)	0.083
Twin pregnancy	37 (9.2%)	21 (5.1%)	0.022
Breech position	142 (35.3%)	182 (44.0%)	0.012
Oligohydramnios	4 (1.0%)	4 (1.0%)	1.000
Prematurity	55 (13.7%)	13 (3.1%)	0.000
Congenital torticollis	10 (2.5%)	17 (4.1%)	0.196
Multiple malformation syndrome	2 (0.5%)	3 (0.7%)	1.000
Congenital foot anomalies	9 (2.2%)	8 (1.9%)	0.795
Ortolani maneuver	21 (5.2%)	13 (3.1%)	0.136
Barlow maneuver	6 (1.5%)	8 (1.9%)	0.632
Galeazzi sign	5 (1.2%)	3 (0.7%)	0.500
Skin fold asymmetry	64 (15.9%)	64 (15.5%)	0.856
Limited abduction	80 (19.9%)	48 (11.6%)	0.001
Limb length discrepancy	5 (1.2%)	2 (0.5%)	0.280
Click	55 (13.7%)	87 (21.0%)	0.006
Double skin fold in the lumbosacral region	2 (0.5%)	16 (3.9%)	0.001

Conversely, referrals for breech presentation, a high-risk factor, increased significantly from 142 to 182 (35.3% to 44.0%, p = 0.012). This indicates that the new protocol effectively directed attention toward this critical risk group.

Notably, referrals for “hip clicks” increased significantly from 55 to 87 (13.7% to 21.0%, p = 0.006), despite this sign not being included as a referral criterion in the protocol. Referrals for limited abduction decreased from 80 to 48 (19.9% to 11.6%, p = 0.001), despite remaining part of the screening protocol.

## Discussion

In concordance with current literature, our cohort demonstrated a predominance of DDH in females, accounting for 60.5% of the diagnosed cases. Various meta-analyses and epidemiological studies confirm that the female sex is one of the strongest isolated risk factors for DDH [8]. In fact, female infants present a significantly higher risk compared to males, with some reports indicating a rate of 75% to 80% of all DDH cases [9,10]. This emphasizes the need for a high index of clinical suspicion when evaluating female newborns, even in the absence of other traditional risk factors.

When evaluating the implementation of the 2020 screening protocol at this hospital, the analysis demonstrated its successful integration into routine clinical practice and its effectiveness in modifying clinical behavior. The significant reduction in referrals for prematurity aligns with current evidence suggesting that prematurity alone is not a strong independent risk factor for DDH. By reducing these unnecessary examinations, the department can better allocate radiology resources.

Simultaneously, the increase in screening for breech presentation from 35.3% to 44.0% represents a favorable trend in protocol compliance. Breech presentation is universally recognized as one of the strongest risk factors for hip dysplasia. Increasing the capture rate of these infants likely prevents missed diagnoses.

However, the implementation of our new protocol resulted in a significant increase in ultrasound referrals motivated by isolated hip “clicks” (21.0%), despite not being included as a formal referral criterion. Extensive evidence demonstrates that isolated high-pitched articular noises, such as “clicks,” are frequently benign sounds arising from the movement of myofascial tissues or ligaments over bony prominences [6,9]. They do not possess clinical significance and are not indicative of true hip instability or DDH [6,10]. The increasing number of referrals for this specific reason underscores the crucial need for continuous medical education, aiding clinicians in confidently differentiating between benign clicks and the pathological “clunks” associated with a dislocated or subluxated hip [10]. It is important to note, however, that our study was not designed to establish the independent diagnostic performance or sensitivity of hip clicks, but rather to highlight how their persistent use as a referral driver can lead to clinical variance.

Our study also reinforces the invaluable role of physical examination. The data demonstrated that a positive Ortolani sign (adjusted OR = 53.2) and a positive Barlow maneuver (adjusted = OR 13.2) are highly significant predictors of DDH. These results agree with international literature and guidelines, where physical examination is a fundamental component of any DDH screening program. The Ortolani maneuver, in particular, remains the most important clinical test for detecting newborn hip dysplasia [10]. Furthermore, the present data support the association between foot deformities and hip dysplasia, reminding clinicians to check the hips carefully in any infant with foot anomalies [10].

Therefore, despite advances in diagnostic strategies, a well-executed clinical assessment remains essential for early identification of at-risk infants and the effectiveness of any DDH screening program.

This study presents certain limitations that must be acknowledged. First, its retrospective, single-center, observational design introduces an inherent risk of selection bias, as we only analyzed infants who were referred for and underwent hip ultrasound, potentially missing infants with DDH who were never referred. Second, there is a risk of intra- and inter-observer variability in both physical examination maneuvers and ultrasound interpretations, which were performed by multiple different clinicians and radiologists. Third, the relatively small absolute number of confirmed DDH cases in our sample and the low frequency of certain positive physical examination findings resulted in wide CIs for several key predictors, such as the Ortolani and Barlow maneuvers. While these associations remain highly statistically significant, the wide intervals reflect a degree of statistical uncertainty regarding the precise magnitude of the estimated effects, meaning these findings should be interpreted with appropriate caution. Fourth, this study did not directly evaluate long-term clinical patient outcomes, such as rates of missed or late-presenting DDH, treatment compliance, or surgical intervention rates; thus, we cannot directly demonstrate whether the modified referral patterns translated into improved clinical outcomes.

## Conclusions

The implementation of a standardized DDH screening protocol successfully modified and standardized referral patterns within the hospital. By significantly reducing unnecessary ultrasound screenings for isolated prematurity, the protocol enabled a potentially more efficient allocation of healthcare resources and optimized clinical pathways. At the same time, it increased the targeted screening coverage for high-risk infants, particularly those with a breech presentation. Despite these improvements, persistent, and even increasing, referrals for benign hip clicks indicate that protocol implementation alone is insufficient. Ongoing medical education is essential to reinforce adherence to screening recommendations and improve the interpretation of physical examination findings, since physical examination remains the most powerful tool for identifying infants at risk. Further prospective studies are required to determine whether these improved referral patterns directly translate into superior patient-level clinical outcomes, such as a reduction in late-diagnosed DDH or surgical intervention rates.
